# Comprehensive Positive Airway Pressure Titration Parameters as Predictors of Therapeutic Adherence in Obstructive Sleep Apnea

**DOI:** 10.3390/life16091462

**Published:** 2026-09-01

**Authors:** Jayoung Oh, Ji Ho Choi

**Affiliations:** 1 Department of Otorhinolaryngology-Head and Neck Surgery, Soonchunhyang University Cheonan Hospital, Soonchunhyang University College of Medicine, 31, Soonchunhyang 6-gil, Dongnam-gu, Cheonan 31151, Republic of Korea; planet90@gmail.com; 2 Department of Otorhinolaryngology-Head and Neck Surgery, Soonchunhyang University Bucheon Hospital, Soonchunhyang University College of Medicine, 170, Jomaru-ro, Bucheon 14584, Republic of Korea

**Keywords:** sleep apnea, obstructive, continuous positive airway pressure, treatment adherence and compliance, regression analysis

## Abstract

**Background:** Whether the physiological response during positive airway pressure (PAP) titration predicts adherence independently of baseline severity is unclear. **Methods:** In 304 patients who underwent manual PAP titration at a tertiary university hospital between 2020 and 2022, adherence was defined by Korean reimbursement criteria (≥4 h/night on ≥70% of nights within any consecutive 30-day period during the initial 90 days). Titration parameters were compared using tests appropriate to each distribution and entered into logistic regression adjusted for age, sex, body mass index and baseline apnea–hypopnea index (AHI), with bootstrap validation. **Results:** Adherent (*n* = 238) and non-adherent (*n* = 66) groups had comparable baseline severity. Three titration parameters differed: AHI reduction rate (*p* = 0.033), lowest oxygen saturation (SpO_2_; *p* = 0.010) and rapid eye movement-sleep proportion (*p* = 0.021). Adding these parameters to a confounder-only model raised the area under the curve from 0.566 to 0.683 (*p* < 0.001; optimism-corrected, 0.632). Adjusted for confounders alone, all three were significant, but with mutual adjustment only the lowest SpO_2_ remained independent (odds ratio, 1.117 per 1%; 95% CI, 1.028–1.214). **Conclusions:** Among the physiological parameters recorded during PAP titration, the lowest SpO_2_ was the most robust predictor of short-term adherence, independent of both baseline disease severity and baseline hypoxemia. Prospective validation is required.

## 1. Introduction

Obstructive sleep apnea (OSA) is a prevalent chronic respiratory disorder characterized by repeated episodes of upper airway collapse during sleep, leading to intermittent hypoxia, sleep fragmentation, and significant cardiovascular, metabolic, and neurocognitive consequences [1,2]. Epidemiological estimates suggest that OSA affects approximately 1 billion people worldwide, with rising obesity rates contributing to its increasing global burden [3,4].

Positive airway pressure (PAP) therapy remains the gold standard treatment for moderate-to-severe OSA, effectively reducing the apnea–hypopnea index (AHI), improving daytime sleepiness, and reducing cardiovascular morbidity and all-cause mortality when used consistently [5,6,7]. However, the therapeutic benefits of PAP are critically dependent on patient adherence, which remains a persistent clinical challenge. Non-adherence rates have remained largely unchanged over the past two decades despite advances in device technology [8], with studies indicating that 30–50% of patients fail to meet the clinically recommended minimum of 4 h per night on at least 70% of nights [9,10]. This underutilization severely attenuates the clinical benefits, leaving patients susceptible to the adverse consequences of untreated OSA.

The determinants of PAP adherence are multifactorial. A comprehensive review has emphasized that adherence is influenced by a complex interplay of patient demographics, disease severity, psychological factors including self-efficacy and outcome expectations [11,12], mask-related comfort issues [13,14], and the quality of early therapeutic experiences [15,16,17]. Notably, adherence patterns appear to be established within the first week of treatment [10,18], highlighting the critical importance of the initial PAP exposure. In-laboratory PAP titration represents this pivotal first encounter, providing the first substantive experience of therapy while generating comprehensive physiological data.

The titration night has a dual character: it is simultaneously a physiological measurement and the patient’s first therapeutic exposure. The response recorded that night is therefore not only a description of respiratory control, but also an objective reflection of the quality of the early treatment experience, and hence of the strength of the initial positive feedback the patient receives. This matters because subjective impressions formed during the first nights of therapy shape subsequent usage trajectories, with adherence patterns largely established within the first week [10,16,17,18]: the magnitude of AHI reduction, the correction of nocturnal oxygenation and the recovery of REM sleep are plausible physiological correlates of the symptomatic relief a patient perceives on waking, and may operate through behavioral mediators such as self-efficacy and outcome expectancy [11,12].

Several studies have explored the relationship between titration night parameters and subsequent adherence. It has been reported that sleep quality during the continuous PAP (CPAP) titration night predicts long-term compliance [19], titration sleep efficiency is associated with both short- and long-term adherence [20], and improvements in sleep parameters during titration polysomnography predict PAP adherence [21]. However, these studies have predominantly employed univariable analyses or examined limited sets of titration metrics without adjusting for potential confounders such as baseline disease severity. A further conceptual challenge concerns the distinction between titration quality and patient-specific treatment responsiveness: a high titration AHI may reflect suboptimal technical execution, rather than an inherent patient characteristic that predicts adherence [22].

Three titration measures are central to this analysis, each for a specific reason. The proportional reduction in AHI measures responsiveness rather than residual severity: two patients may end the night with the same titration AHI of 5 events/h, one having started from 20 and the other from 60, and the absolute value treats them as equivalent, while the proportional reduction records that the second responded far better [21,22]. The lowest SpO_2_ during titration is not baseline hypoxemia restated; measured under an effective pressure, it shows how much desaturation pressure has failed to correct, and may point to causes that pressure alone cannot fix, such as reduced ventilatory reserve, obesity hypoventilation, coexisting lung disease or marked positional dependence [23]. REM sleep, finally, is the state in which the airway is most collapsible, dilator muscle tone being lowest and respiratory events longest and most desaturating; its return under pressure, therefore, shows that the pressure is adequate where adequacy is hardest to achieve [24,25,26].

Building on these considerations, we set out to characterize a comprehensive set of titration parameters in relation to short-term adherence, to determine whether they add prognostic information beyond baseline demographic characteristics and disease severity, and to assess the stability of the resulting model by internal validation with bootstrap and cross-validation, in accordance with current prediction-model reporting guidelines [27,28].

## 2. Materials and Methods

### 2.1. Study Design and Participants

This was a retrospective cohort study conducted at a tertiary university hospital in Korea. We analyzed 304 consecutive patients diagnosed with OSA who underwent overnight PAP titration between January 2020 and December 2022. Inclusion criteria were (1) age ≥ 20 years; (2) diagnosis of OSA confirmed by standard polysomnography (AHI ≥ 5 events/h with symptoms or AHI ≥ 15 events/h, regardless of symptoms), according to the *International Classification of Sleep Disorders, Third Edition, Text Revision* [29]; (3) consent to PAP therapy; (4) successful completion of overnight PAP titration; and (5) availability of PAP adherence data during the initial 90-day monitoring period.

This study conformed to the ethical guidelines of the 1975 Declaration of Helsinki and was approved by the Institutional Review Board (approval no. 2025-05-004). The requirement for informed consent was waived due to the retrospective nature of the study.

### 2.2. Adherence Definition

PAP adherence was defined according to Korean national health-insurance reimbursement guidelines as device usage of ≥4 h per night on ≥70% of nights within any consecutive 30-day period during the initial 90-day treatment window [30,31]. This criterion was selected because it constitutes the operationally relevant threshold in the Korean healthcare system, where insurance coverage of PAP therapy is contingent upon meeting this standard. Patients were classified into adherence and non-adherence groups, based on this criterion.

### 2.3. Data Collection

Baseline demographic and clinical data collected at enrollment included age, sex, body mass index (BMI), neck circumference, and waist-to-hip ratio. Subjective sleepiness was assessed using the Epworth Sleepiness Scale, OSA risk was evaluated with the STOP-Bang questionnaire, and sleep quality was measured using the Pittsburgh Sleep Quality Index.

All participants underwent full diagnostic polysomnography using a digitized system (Embla N7000; Natus Medical Inc., San Carlos, CA, USA) recording electroencephalography, electrooculography, electromyography (chin and bilateral anterior tibialis), electrocardiography, nasal/oral airflow (thermistor and nasal pressure transducer), thoracoabdominal effort (inductance plethysmography), pulse oximetry, body position, and snoring. All studies were manually scored by a certified sleep technologist in accordance with the *American Academy of Sleep Medicine (AASM) Manual for the Scoring of Sleep and Associated Events* [32] and were subsequently reviewed by a board-certified sleep physician.

### 2.4. PAP Titration Protocol

Overnight manual PAP titration was performed using the same polysomnographic montage as the diagnostic study, following the AASM clinical guidelines for manual titration of PAP [22]. The optimal pressure was defined as the lowest effective pressure that eliminated obstructive respiratory events, including apneas, hypopneas, respiratory effort-related arousals (RERAs), and snoring, in all sleep positions and stages, with particular attention to rapid eye movement (REM) sleep in the supine position. Titration outcomes were then classified according to these AASM criteria as optimal (respiratory disturbance index [RDI] < 5 events/h for ≥15 min, including supine REM sleep at the selected pressure), good (RDI ≤ 10 events/h or a ≥50% reduction from baseline), adequate (≥75% reduction in RDI from baseline) or unacceptable [22]. One definitional point follows from this. These criteria are expressed in terms of the RDI, whereas the pre-planned subgroup analysis reported here used a titration AHI threshold of <5 events/h. The two are not interchangeable, since the RDI additionally incorporates RERAs and is therefore systematically higher; RERA scoring in the routine clinical titration reports available for this retrospective cohort was too incomplete for the RDI-based criterion to be applied reliably. We therefore describe this subgroup throughout as having achieved an AHI-based approximation of AASM-defined optimal titration, rather than equating it with the formal RDI-based category. All titration respiratory-event indices used in the analyses were calculated from the entire titration night, encompassing every pressure level applied during the study.

### 2.5. Titration Variables

The following titration-derived parameters were extracted: (1) titration AHI, RDI, and oxygen desaturation index (ODI) during the titration study; (2) lowest oxygen saturation (SpO_2_) during titration; (3) AHI reduction rate, calculated ([baseline AHI − titration AHI]/baseline AHI) × 100, quantifying the proportional improvement in respiratory events relative to diagnostic severity; (4) titration arousal indices (total, respiratory, and spontaneous); (5) RERA index during titration; (6) supine AHI during titration; (7) titration sleep architecture (N1, N2, N3, and REM sleep proportions); (8) titration sleep efficiency; and (9) optimal titration pressure (cmH_2_O). The AHI reduction rate was chosen as a primary variable because it normalizes the titration event burden to baseline disease severity, thereby distinguishing individual treatment responsiveness from absolute titration event counts that may be confounded by initial disease severity.

### 2.6. Statistical Analysis

Continuous variables are reported as mean ± standard deviation and categorical variables as *n* (%); categorical variables were compared by χ^2^ tests. For continuous variables the distribution was examined by Shapiro–Wilk testing with histograms and quantile–quantile plots, and the test chosen to match the results: independent-samples *t*-tests where normality was approximately satisfied and Mann–Whitney U tests where it was not. Because the two groups differed markedly in size (238 vs. 66) and the variances of several variables were unequal, Welch’s correction was applied to all *t*-tests, rather than pre-testing for homogeneity of variance and selecting between the pooled and unpooled forms.

Univariable logistic regression was performed for each titration parameter, reported as odds ratios (ORs) with 95% confidence intervals (CIs) and areas under the receiver operating characteristic curve (AUCs). ORs are expressed per clinically meaningful increment (e.g., per 5 events/h for AHI, per 10% for AHI reduction rate, per 5% for SpO_2_) [33]. These analyses served as an exploratory screen to identify candidates for the multivariable model, and were not corrected for multiplicity.

The multivariable model included the titration variables with significant univariable associations (*p* < 0.05), adjusted for the a priori confounders of age, sex, BMI and baseline AHI. The AHI reduction rate was chosen over titration AHI to limit collinearity with baseline AHI, and collinearity was checked with variance inflation factors, a value of 5 or more being taken to indicate a problem. To test whether the titration parameters added anything as a group, this model was compared with one containing the confounders alone, using a likelihood-ratio test and the change in AUC. Because adjusting correlated measures for one another can hide associations that are real, each parameter was also modeled with the confounders alone, and the parameters were then added one at a time to see how much each improved discrimination; these sequential analyses were exploratory, and not pre-specified.

Internal validation used 500-iteration bootstrapping to estimate optimism and derive an optimism-corrected AUC [28,34], and repeated (20 × 5-fold) stratified cross-validation to estimate out-of-sample performance. Calibration was assessed graphically and quantified by the calibration slope and intercept, obtained by regressing the observed outcome on the model’s linear predictor, and by the Brier score; all three were bootstrap-corrected for optimism. No a priori sample-size calculation was performed; all consecutive eligible patients treated during the study period were analyzed.

A planned subgroup analysis compared adherence between patients who achieved an AHI-based approximation of AASM-defined optimal titration (titration AHI < 5 events/h) and those who did not; the power of this comparison was evaluated post hoc. Two further analyses were added. The AHI reduction rate was divided into pre-specified categories (<70%, 70–85%, 85–95%, ≥95%) and adherence calculated in each, with a Cochran–Armitage test for trend. The model, which was restricted to the three principal titration predictors and adjusted for the four confounders, was then refitted within the optimal- and suboptimal-titration subgroups, and interaction terms were used to test whether the effects differed between them.

All analyses were performed using R software version 4.4.1 (R Foundation for Statistical Computing, Vienna, Austria) and Python 3.11 (Python Software Foundation, Wilmington, DE, USA) with scikit-learn. Statistical significance was defined as a two-sided *p*-value of less than 0.05.

## 3. Results

### 3.1. Baseline Characteristics

Of the 304 participants, 238 (78.3%) met the adherence criteria and 66 (21.7%) were classified as non-adherent. Table 1 presents the comprehensive comparison of demographic, clinical, polysomnographic, and PAP titration characteristics between the two groups.

Baseline demographic and clinical characteristics were highly comparable between groups. Mean age (49.5 ± 12.2 vs. 48.7 ± 14.6 years; *p* = 0.683), sex distribution (84.9% vs. 81.8% male; *p* = 0.681), BMI (28.8 ± 4.8 vs. 29.0 ± 5.4 kg/m^2^; *p* = 0.841), and subjective sleepiness scores (ESS, 9.3 ± 5.1 vs. 8.9 ± 5.1; *p* = 0.581) did not differ significantly. Baseline polysomnographic severity indices were also comparable, including diagnostic AHI (44.3 ± 25.4 vs. 39.0 ± 24.5 events/h; *p* = 0.126), RDI (49.0 ± 23.5 vs. 44.2 ± 22.6; *p* = 0.130), ODI (41.6 ± 25.8 vs. 37.9 ± 24.5; *p* = 0.281), and lowest SpO_2_ (77.5 ± 8.8% vs. 77.9 ± 9.0%; *p* = 0.720). Baseline sleep architecture did not differ significantly (N1, *p* = 0.051; N2, *p* = 0.053).

### 3.2. PAP Titration Parameters

In contrast to the comparable baseline profiles, three titration parameters differed significantly between groups (Table 1). The non-adherence group showed a lower AHI reduction rate (76.3 ± 29.2% vs. 86.8 ± 13.3%; *p* = 0.033), a lower lowest SpO_2_ during titration (87.55 ± 5.29% vs. 89.40 ± 4.09%; *p* = 0.010) and a lower proportion of REM sleep (18.0 ± 7.7% vs. 20.5 ± 7.3%; *p* = 0.021). The titration event indices did not differ: titration AHI (*p* = 0.133), titration RDI (*p* = 0.204), titration supine AHI (*p* = 0.129) and the respiratory arousal index (*p* = 0.108); nor did titration pressure (8.71 ± 2.72 vs. 8.62 ± 2.89 cmH_2_O; *p* = 0.808) or the total arousal index (*p* = 0.747). These event indices were markedly right-skewed, with standard deviations approaching or exceeding their means and normality rejected on Shapiro–Wilk testing (*p* < 0.001 for all), and were therefore compared non-parametrically.

### 3.3. Univariable Logistic Regression Analysis

Seven of the eight titration parameters were significantly associated with adherence in univariable logistic regression (Table 2), the exception being titration ODI (*p* = 0.071). The strongest was the AHI reduction rate (OR, 1.305 per 10% increase; *p* < 0.001), the only parameter to meet the Bonferroni-adjusted threshold of α = 0.00625. However, no single parameter discriminated well (AUC, 0.54–0.61). Baseline AHI was not significantly associated with adherence (OR, 1.009 per 1 event/h; 95% CI, 0.997–1.020; *p* = 0.132), but was retained as an a priori confounder because the AHI reduction rate is defined relative to it.

### 3.4. Multivariable Logistic Regression

The multivariable model included four titration parameters (AHI reduction rate, titration lowest SpO_2_, respiratory arousal index and REM sleep proportion) adjusted for age, sex, BMI and baseline AHI (Table 3). When all four were entered together, the titration lowest SpO_2_ was the only parameter that remained independently associated with adherence (OR, 1.117 per 1%; 95% CI, 1.028–1.214; *p* = 0.009; Table 3). The AHI reduction rate (*p* = 0.361) and the REM sleep proportion (*p* = 0.062) were attenuated in that model, although each was significant when modeled separately with the confounders alone: AHI reduction rate (OR, 1.288 per 10%; 95% CI, 1.111–1.493), lowest SpO_2_ (OR, 1.921 per 5%; 95% CI, 1.342–2.751) and REM sleep proportion (OR, 1.271 per 5%; 95% CI, 1.034–1.563). The REM association reached significance once the respiratory arousal index was dropped from the model (*p* = 0.047), and the null result for the respiratory arousal index was unchanged after logarithmic transformation of this markedly skewed variable (*p* = 0.669). As a group, the titration parameters added substantially to the model: including them raised the apparent AUC from 0.566 to 0.683 (likelihood-ratio χ^2^(4) = 22.07; *p* < 0.001). None of the adjustment variables were significant, and variance inflation factors were all below 3; substituting the absolute titration AHI for the AHI reduction rate raised the value for that term to 20.46.

Added one at a time to the confounder model, the titration lowest SpO_2_ gave the largest gain in discrimination (AUC, 0.566 to 0.651), with the other three parameters each reaching 0.63 or less; adding the REM sleep proportion on top of the lowest SpO_2_ improved the model further (AUC, 0.680; *p* = 0.014), whereas the AHI reduction rate and the respiratory arousal index added little. The AHI reduction rate lost significance specifically because of the respiratory arousal index, with which it is strongly correlated (*r* = −0.68): it remained significant when the lowest SpO_2_ was added to the model (*p* = 0.046), but not when the respiratory arousal index was added (*p* = 0.132). The titration lowest SpO_2_ was independent of the baseline value, which was itself unrelated to adherence (*p* = 0.471); the titration value remained significant after adjustment for it (OR, 1.937 per 5%; *p* < 0.001).

**Figure 1:**
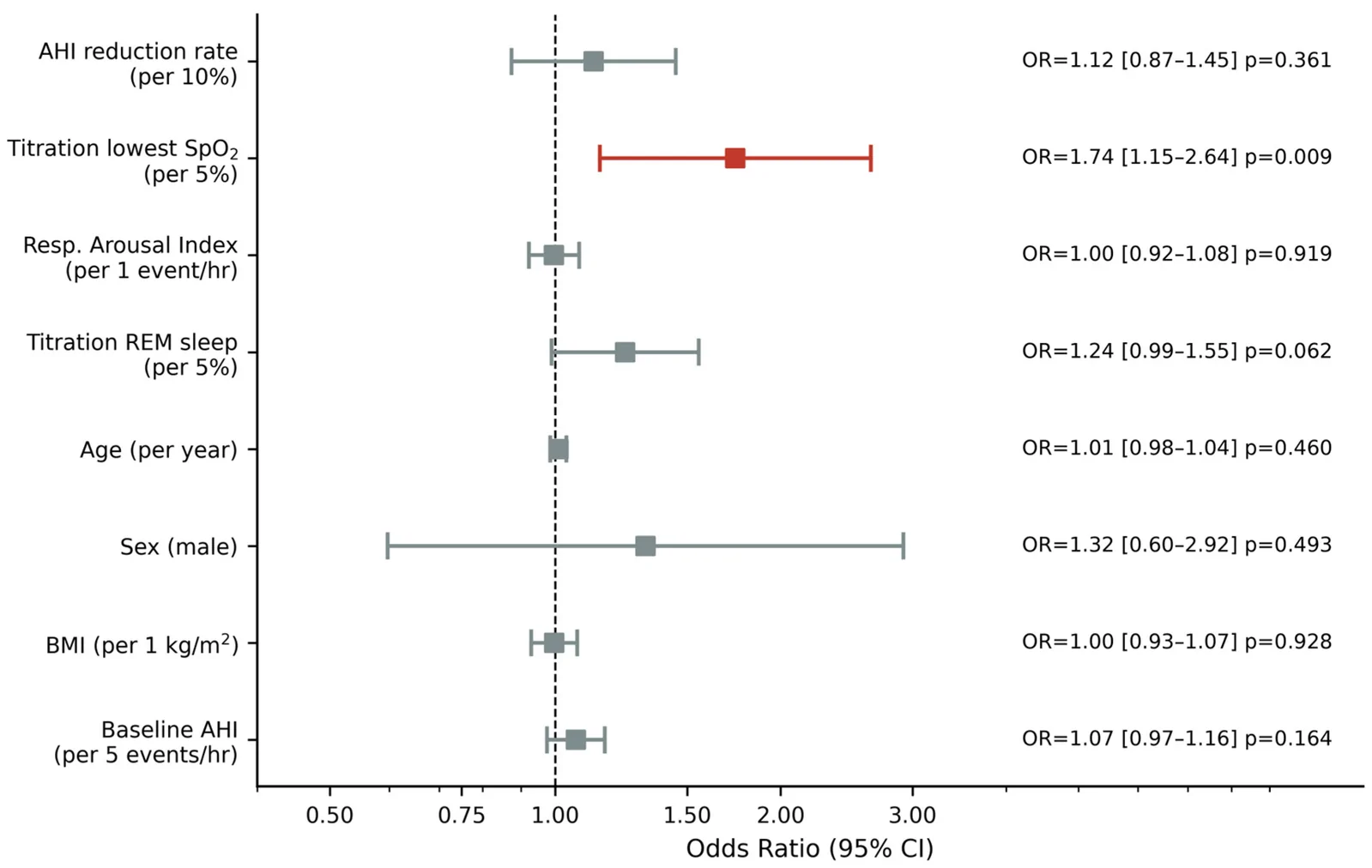
Forest plot of multivariable logistic regression results. Odds ratios (ORs) with 95% confidence intervals (CIs) for each predictor variable. Red markers indicate statistically significant associations (*p* < 0.05). Gray markers indicate non-significant associations. AHI, apnea–hypopnea index; SpO_2_, oxygen saturation; Resp., respiratory; REM, rapid eye movement; BMI, body mass index.

Figure 1 displays the model as a forest plot with odds ratios rescaled to clinically meaningful increments; the titration lowest SpO_2_ provides the only interval excluding unity (OR, 1.74 per 5%; 95% CI, 1.15–2.64). Because any ranking by odds ratio depends on the increment chosen, these values should not be read as a ranking of importance.

### 3.5. Model Discrimination and Internal Validation

**Figure 2:**
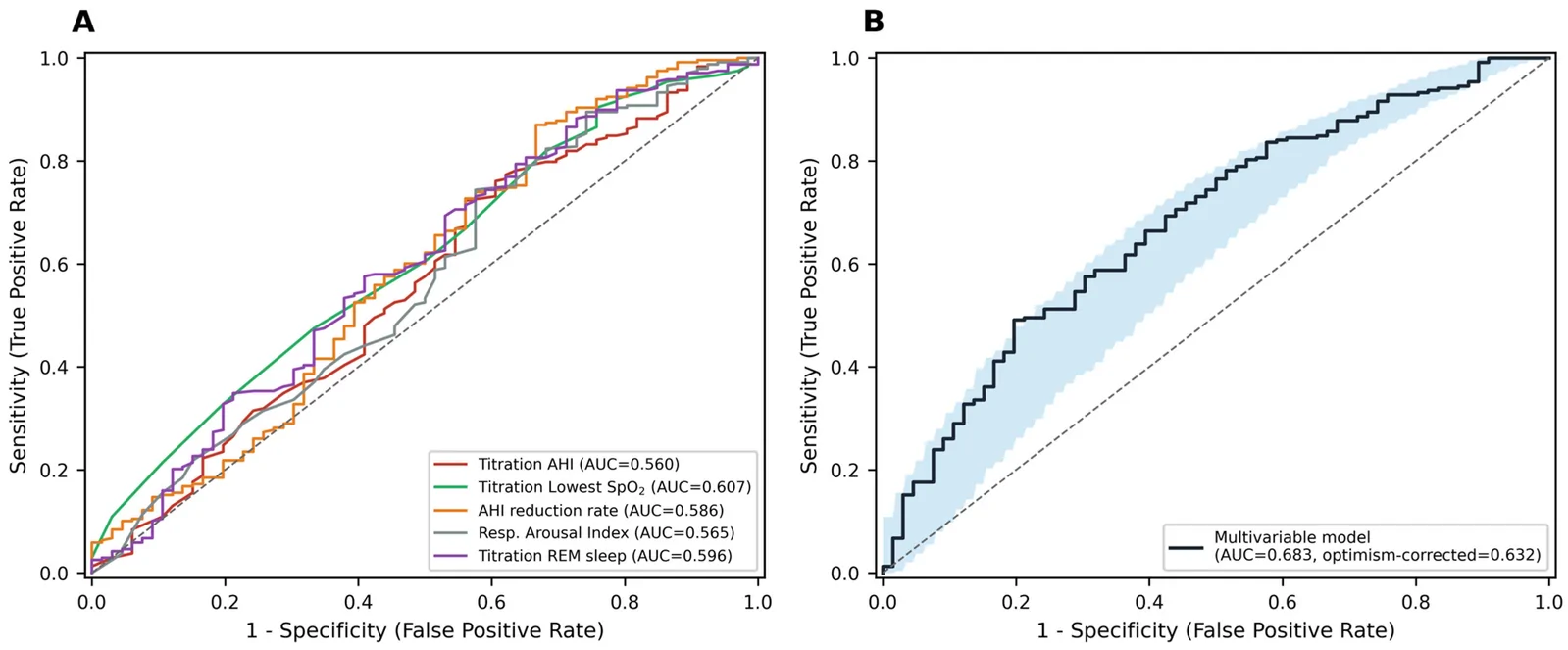
Receiver operating characteristic (ROC) curves. (**A**) Univariable ROC curves for individual titration parameters. (**B**) Multivariable model ROC curve with 95% bootstrap confidence interval (shaded area). AHI, apnea–hypopnea index; AUC, area under the ROC curve; SpO_2_, oxygen saturation; Resp., respiratory; REM, rapid eye movement.

**Figure 3:**
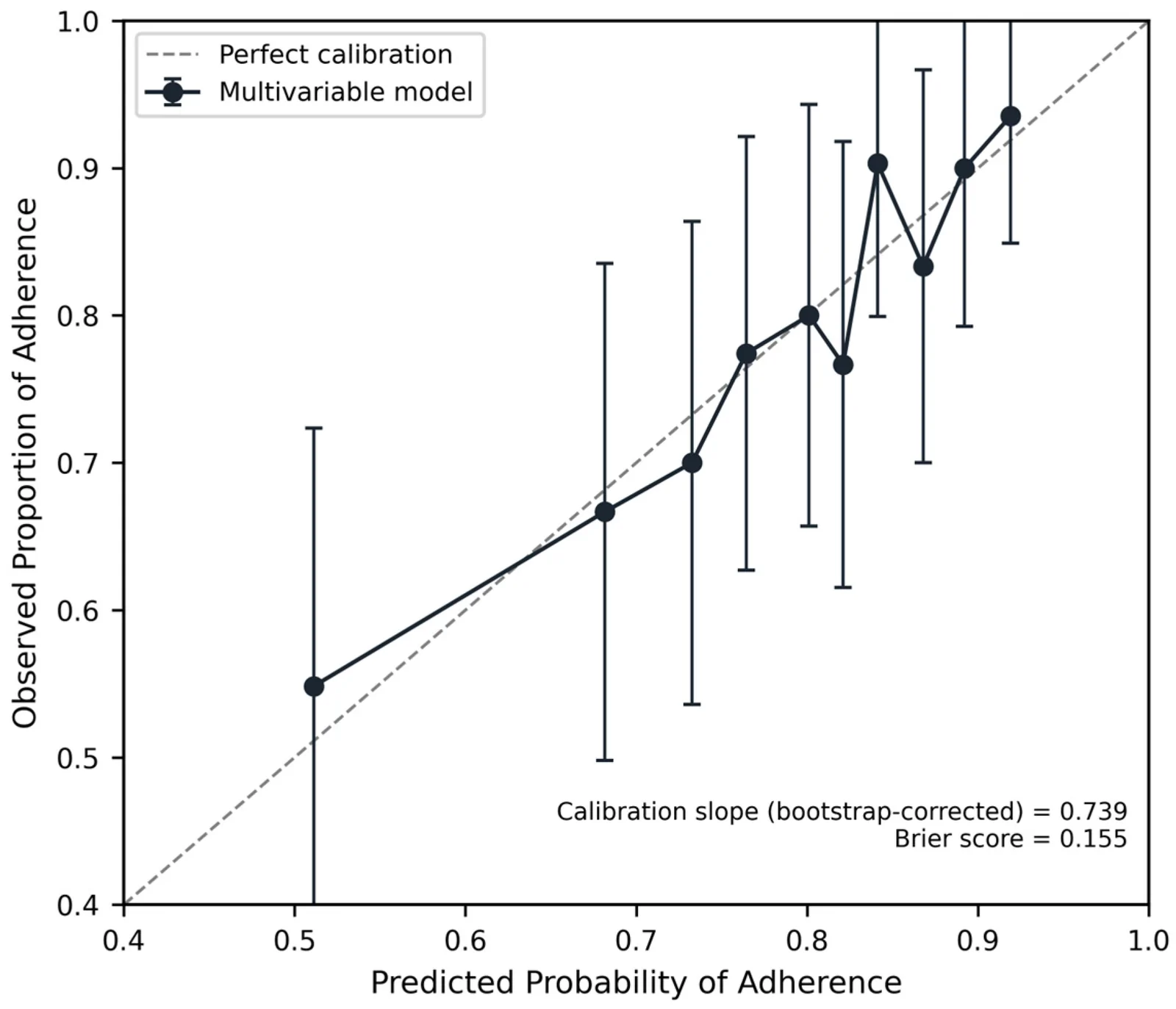
Calibration plot for the multivariable prediction model. Predicted probability of adherence versus observed proportion of adherence across quantile-based groups. The dashed diagonal line represents perfect calibration. The bootstrap-corrected calibration slope was 0.739 and the apparent Brier score 0.155.

The multivariable model yielded an apparent AUC of 0.683, compared with 0.566 for a model containing age, sex, BMI and baseline AHI alone. Internal validation using 500-iteration bootstrap estimated an optimism of 0.051, giving an optimism-corrected AUC of 0.632 (bootstrap 95% CI, 0.563–0.700). Repeated (20 × 5-fold) stratified cross-validation gave a mean AUC of 0.607 ± 0.023, consistent with the bootstrap estimate, and indicating modest but stable discrimination. Figure 2 presents the receiver operating characteristic curves for the individual titration variables (Figure 2A) and for the multivariable model with bootstrap 95% confidence intervals (Figure 2B). The calibration plot (Figure 3) showed reasonable agreement between predicted and observed adherence probabilities, with an apparent Brier score of 0.155 (optimism-corrected, 0.166). The calibration slope is 1.000 by construction in the sample used to fit the model; after bootstrap correction it fell to 0.739. An optimism-corrected AUC of 0.632 means that, given one adherent and one non-adherent patient chosen at random, the model ranks the adherent patient higher about 63% of the time. This is not enough for the model to be used on its own to make decisions about an individual patient, but is compatible with its use as one component of a broader risk assessment that also takes account of psychological, social and device-related factors.

### 3.6. Adherence According to the Magnitude of AHI Reduction

Patients were grouped by AHI reduction rate into four pre-specified categories. Adherence was 56.1% (23/41) below a 70% reduction, 81.4% (48/59) at 70–85%, 83.6% (107/128) at 85–95% and 78.9% (60/76) at ≥95% (Cochran–Armitage test for trend, *z* = 2.43; *p* = 0.015). The pattern is a threshold, rather than a gradient: adherence was much lower only in the patients whose AHI fell by less than 70%, and was essentially flat across the three higher categories, with no further gain beyond a 95% reduction.

### 3.7. Optimal Titration Subgroup Analysis

We compared adherence rates between patients who achieved an AHI-based approximation of AASM-defined optimal titration (titration AHI < 5 events/h; *n* = 187) and those with suboptimal titration (AHI ≥ 5; *n* = 117). No significant difference in adherence was detected between the two groups (80.7% vs. 74.4%; χ^2^ = 1.373; *p* = 0.241). The comparison was underpowered: with these group sizes, the probability of detecting a difference as small as the observed 6.3 percentage points was only 25.1%, and a difference of 13.6 percentage points would have been needed for 80% power.

**Figure 4:**
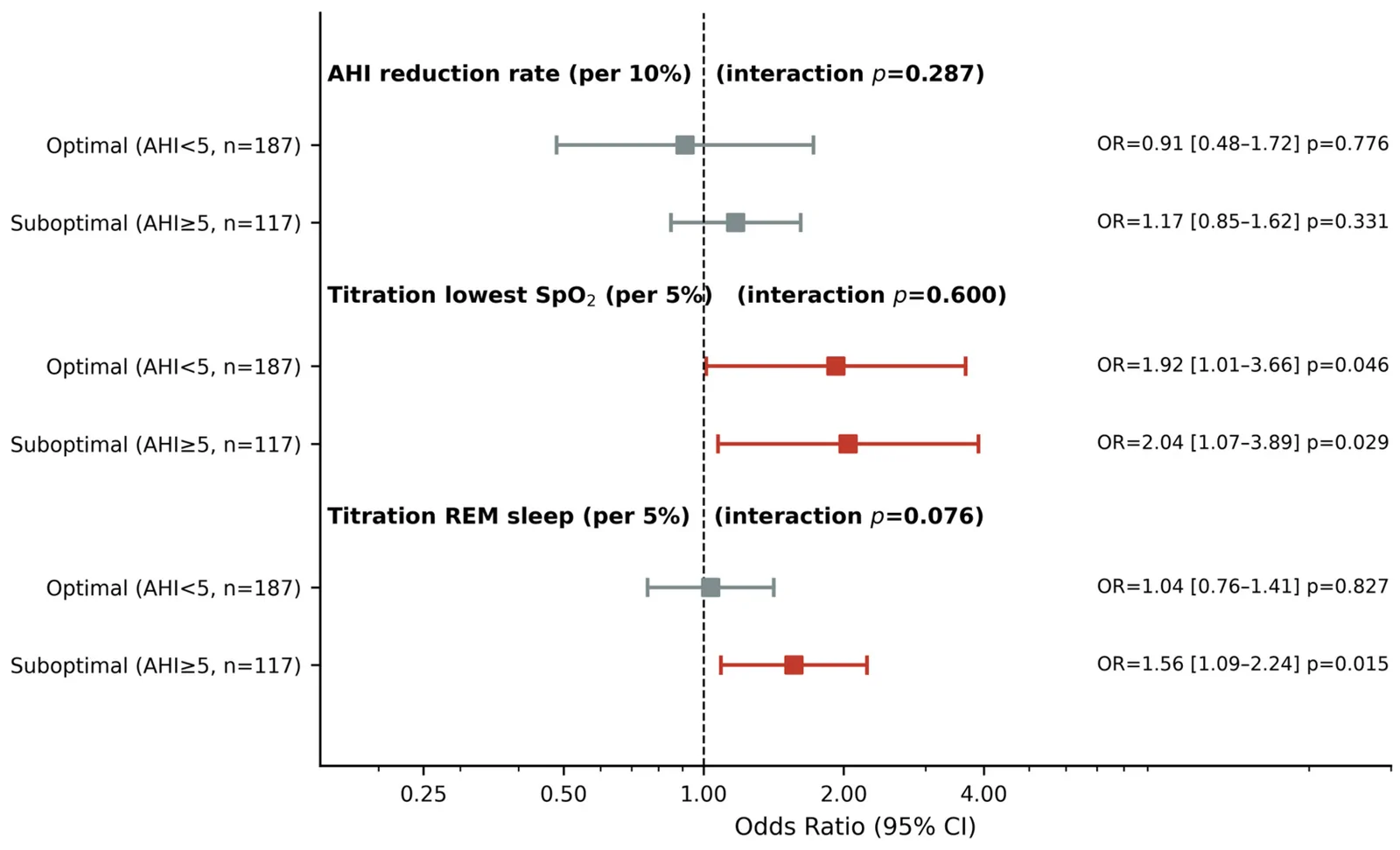
Stratified forest plot of the three principal titration predictors, refitted separately within the optimal-titration (titration AHI < 5 events/h, *n* = 187) and suboptimal-titration (*n* = 117) subgroups, each adjusted for age, sex, body mass index and baseline AHI. Odds ratios are shown per clinically meaningful increments with 95% confidence intervals, together with the *p*-value for the corresponding multiplicative interaction term. Red markers indicate statistically significant associations (*p* < 0.05). Gray markers indicate non-significant associations. AHI, apnea–hypopnea index; SpO_2_, oxygen saturation; REM, rapid eye movement.

To examine whether the predictive effects held in both subgroups, the multivariable model restricted to the three principal titration predictors was refitted separately within each subgroup (Figure 4). The titration lowest SpO_2_ was significantly associated with adherence in both strata, and with almost identical effect size (optimal titration: OR, 1.92 per 5%; 95% CI, 1.01–3.66; *p* = 0.046; suboptimal titration: OR, 2.04 per 5%; 95% CI, 1.07–3.89; *p* = 0.029; interaction *p* = 0.600). The REM sleep proportion was significant only in the suboptimal-titration subgroup (OR, 1.56 per 5%; 95% CI, 1.09–2.24; *p* = 0.015) and null in the optimal-titration subgroup (OR, 1.04; *p* = 0.827; interaction *p* = 0.076). The AHI reduction rate was not significant in either stratum (interaction *p* = 0.287).

## 4. Discussion

In this retrospective cohort of 304 patients with OSA, the physiological response recorded during PAP titration was associated with short-term adherence independently of baseline disease severity and demographic characteristics. Adding the four titration parameters examined (AHI reduction rate, titration lowest SpO_2_, respiratory arousal index and REM sleep proportion) to a model containing age, sex, BMI and baseline AHI increased the apparent AUC from 0.566 to 0.683 (*p* < 0.001), with an optimism-corrected AUC of 0.632. When each parameter was adjusted for these confounders, but not for one another, the AHI reduction rate, the titration lowest SpO_2_ and the REM sleep proportion were each significantly associated with adherence; when all four were entered together, only the lowest SpO_2_ retained independent significance. Sequential modeling indicated that the prognostic information was concentrated in oxygenation, rather than distributed evenly among the parameters: the lowest SpO_2_ produced the largest single gain in discrimination, the REM sleep proportion contributed additional information, and the AHI reduction rate contributed little, once the respiratory arousal index, with which it was strongly correlated, had been taken into account.

These findings both extend and qualify the existing literature on titration-night predictors of adherence. Sleep quality and sleep efficiency during titration have been reported to predict subsequent CPAP use [19,20], as has improvement in sleep parameters during titration polysomnography [21]; those analyses were univariable or minimally adjusted. Our univariable results are concordant with those reports, but the multivariable analysis shows that most of the individual associations do not survive mutual adjustment, which suggests that earlier single-parameter findings may reflect a shared underlying signal, rather than independent effects. Nocturnal hypoxemia indices measured during diagnostic polysomnography have been identified as predictors of adherence over 10 years [23]; in the present cohort, the baseline lowest SpO_2_ was unrelated to adherence, whereas the titration lowest SpO_2_ was independently predictive, indicating that the oxygenation response to treatment, rather than the severity of untreated hypoxemia, carries the prognostic weight. Our group has previously reported associations between short-term adherence and demographic and diagnostic sleep-study variables [30] and titration sleep-continuity measures [31] in Korean cohorts; the present analysis adds respiratory and oxygenation parameters and, by adjusting them for one another, identifies which retain independent value. Conventional titration-outcome categories have been found to have limited predictive value for long-term adherence [35], consistent with our observation that a binary classification of titration success did not distinguish adherent from non-adherent patients.

The adherence rate of 78.3% observed here lies at the upper end of the 70–76% reported in Korean cohorts [30,31,36] and above the 75% reported in a large real-world analysis [18]. The Korean reimbursement framework, which requires compliance to be demonstrated within 90 days for continued coverage, provides a financial incentive for early use that is absent in many other healthcare systems, and this difference should be considered when comparing adherence rates across countries.

A recurring concern in this field is that associations between titration parameters and adherence may be tautological, in that suboptimal titration leads to an inappropriate pressure prescription and, therefore, to poor adherence [22]. The present data argue against a purely technical explanation. Adherence did not differ significantly between patients who did and did not achieve a titration AHI < 5 events/h (80.7% vs. 74.4%; *p* = 0.241), although this comparison had only 25.1% power, and is not informative in isolation. More persuasive is the stratified analysis, in which the association between the lowest SpO_2_ and adherence was almost identical among patients whose titration was technically successful (OR, 1.92 per 5%) and among those in whom it was not (OR, 2.04 per 5%). An association of unchanged magnitude within the technically successful stratum cannot be attributed to technical failure. Whether the same applies to the AHI reduction rate and the REM sleep proportion, which were not consistently significant across strata, remains uncertain.

The independent association between the titration lowest SpO_2_ and adherence may be explained in two ways, which are not mutually exclusive. First, effective correction of nocturnal oxygenation on the first therapeutic night may reinforce the patient’s perception that treatment is effective, and perceived benefit is an established determinant of early adherence [11,12]. Second, a low SpO_2_ persisting under an adequately titrated pressure may identify patients in whom hypoxemia is not purely obstructive in origin. Residual desaturation despite pressure support can reflect coexisting chronic lung disease, obesity hypoventilation, reduced ventilatory reserve or marked positional dependency, conditions associated with greater treatment discomfort, slower symptomatic benefit and a greater need for pressure or interface adjustment. On this interpretation, the titration lowest SpO_2_ reflects pathophysiological complexity in addition to the quality of the first therapeutic night, which would account for the information it carries beyond measures of event suppression. Data on pulmonary comorbidity, awake capnography and body position were not systematically available, and the two explanations could not be distinguished in this cohort.

The REM sleep proportion achieved during titration was associated with adherence, although the association was not robust: it did not reach significance in the fully adjusted model (*p* = 0.062), reached significance only when the respiratory arousal index was removed (*p* = 0.047), and, in the stratified analysis, was confined to patients with suboptimal titration. The underlying mechanism is, nevertheless, plausible. REM sleep is the state in which the upper airway is most collapsible, since dilator muscle tone is lowest and arousal and chemoreflex responses are attenuated, so that respiratory events are longest and most desaturating and REM periods are repeatedly terminated [24,25,26]. The reappearance of REM sleep during titration, therefore, indicates that the selected pressure is adequate under the most demanding conditions, which is the objective of manual titration [22]. That the association was confined to patients with suboptimal titration is consistent with this account, since achieving REM sleep conveys little additional information once respiratory events have already been abolished. REM rebound is also perceptible to patients, its reported correlates including increased dream recall, a sense of deeper sleep, and reduced morning grogginess [25], so it may be one of the few polysomnographic variables that corresponds to the patient’s own experience on the following morning.

The AHI reduction rate was the strongest predictor in univariable analysis and the only parameter to meet a Bonferroni-adjusted threshold, and it remained significant after adjustment for age, sex, BMI and baseline AHI (OR, 1.288 per 10%). It did not retain independence when the other titration measures were included, and is therefore best regarded as a robust marker of the overall titration response, rather than as an independent predictor. Its principal advantage is conceptual. Patients with more severe OSA retain more events even after an adequate titration, so the absolute titration AHI remains confounded by baseline severity, whereas the proportional reduction expresses the treatment response relative to each patient’s own starting point; the collinearity diagnostics supported this choice, the variance inflation factor rising from 2.91 for the proportional reduction to 20.46 when the absolute titration AHI was substituted. This is consistent with the growing recognition that treatment response, rather than absolute disease control, predicts behavioral outcomes [15,21].

The clinical relevance of proportional, rather than absolute, improvement may lie in the perception of relative gain. A titration AHI of 5 events/h represents a 75% improvement for a patient with a baseline AHI of 20 and a 92% improvement for a patient with a baseline of 60; the physiological endpoint is identical, but the symptomatic change perceived by the patient is not. If early adherence is driven substantially by the reinforcement value of the first therapeutic experience [16,17], a measure of proportional change would be expected to track that experience more closely than a measure of the residual state, in keeping with cognitive models in which perceived benefit and outcome expectancy determine the self-efficacy that sustains use [11,12,37,38]. The threshold pattern observed here, in which adherence was reduced only below a 70% reduction and did not increase further beyond 95%, is compatible with this interpretation, although it requires confirmation in a prospective cohort with patient-reported symptom measures obtained on the morning after titration.

The respiratory arousal index was associated with adherence in univariable analysis (*p* = 0.011) but showed no association after multivariable adjustment (*p* = 0.919), and the between-group difference did not survive non-parametric testing (*p* = 0.108). The most likely explanation is mediation, rather than absence of relevance: sleep fragmentation during the first PAP exposure is itself a consequence of residual respiratory events, so that once event suppression has been accounted for, the arousal index carries no additional prognostic information. Its inclusion in the model was not neutral, since its correlation with the other titration measures reduced the REM sleep coefficient below significance.

An optimism-corrected AUC of 0.632 is modest, but comparable with previously reported prediction models based on pre-treatment data. Reported values are 0.55–0.65 for demographic and polysomnographic predictors [39] and 0.60–0.70 for pre-treatment variables [40], and machine learning models have rarely exceeded 0.65 when restricted to baseline features [41]; an AUC of 0.73 has been achieved only when early device-use data were included [42]. Adherence therefore appears to be a behavioral phenotype with a substantial component that pre-treatment physiological measurement cannot capture. Together with a bootstrap-corrected calibration slope of 0.739, these figures indicate that titration-derived parameters are better suited to risk stratification at the group level than to prediction in individual patients.

Models restricted to physiological variables, including ours, omit psychological determinants of adherence that are well established. Self-efficacy and outcome expectations [11,38], coping style and health locus of control [43] and pre-treatment health beliefs [37] have all been shown to predict CPAP use, and a systematic review has confirmed that personality traits and pre-treatment beliefs predict adherence consistently across populations [44]. An integrative framework has been proposed in which psychological and physiological factors interact, with the initial therapeutic experience modulating subsequent cognitive appraisal [12]. The present findings are compatible with that framework, since the physiological response during titration may shape the patient’s first perception of treatment. Studies combining titration-derived physiological markers with psychological assessment represent a logical next step.

The importance of the early treatment experience is well established. First-week use strongly predicts subsequent adherence, defining an early critical window [16], and a brief survey administered immediately after the titration night has been shown to predict later use [17]. Pharmacologically improving sleep quality during the titration night improves both titration outcomes and subsequent compliance [45,46], providing interventional evidence that the titration experience influences adherence, rather than merely marking it.

Although this study was not designed to derive cut-points and no threshold reported here is validated, three low-cost measures follow from the observed associations and could be evaluated prospectively. First, the AHI reduction rate could be added as a routine field to the titration report; it requires no additional measurement, being calculated from two values that are already recorded, and makes the magnitude of the therapeutic response explicit, rather than leaving it to be inferred from the residual AHI. Second, patients whose titration response resembles that of the non-adherent group in this cohort (an AHI reduction below approximately 70%, at which the observed adherence rate was 56.1%; a titration lowest SpO_2_ persistently below 88%; or a REM sleep proportion below approximately 15%) could be identified for earlier follow-up: for example, a structured telephone review within the first week and review every two weeks, rather than monthly during the first three months, so that mask, pressure and comfort problems are addressed while usage habits are still forming [10,16,18]. These figures are descriptive anchors derived from the present cohort, rather than validated thresholds, and the evidence supporting the SpO_2_ and REM anchors is weaker than that supporting the AHI reduction anchor. Third, the same data may be used in communication with patients: presenting the magnitude of the individual response, for example, a fall in AHI from 50 to 5 events/h corresponding to a 90% reduction, converts an abstract measurement into explicit feedback of the kind that supports self-efficacy [11,12,37,38]. Whether such feedback improves usage remains unproven, since the evidence that educational and behavioral interventions increase CPAP use is of low certainty [47], and any risk-stratified pathway based on titration parameters would require prospective evaluation before adoption.

Several limitations should be considered. The modest discriminative performance of the model reflects the fact that PAP adherence is a behavioral outcome driven substantially by psychological, social and device-related factors that no single-night physiological assessment can capture [11,12,15].

Variables that plausibly influence both the titration result and adherence were unavailable, including mask leak, which inflates the titration AHI and degrades sleep quality and is therefore a potential confounder of several predictors at once [13,14,48]; mask type and fit [49]; patient-reported comfort [17]; psychological constructs [11,37,38,43,44]; comorbid insomnia [50,51]; and the pulmonary and positional data needed to test our interpretation of the lowest SpO_2_. RERA scoring was also too incomplete for the RDI-based AASM definition of optimal titration to be applied, and titration event indices were calculated across the entire night, including subtherapeutic periods [22]. Prospective studies should integrate physiological, psychological and technical predictors, rather than any one domain alone.

Adherence was defined by Korean reimbursement criteria within a 90-day window, which is objective in this setting but linked to a financial incentive for early use that many healthcare systems lack, so neither the adherence rate nor, necessarily, the strength of the associations can be assumed to transfer [30]. The outcome is also short-term and behavioral; whether these parameters predict sustained use beyond one year, cardiovascular events, quality of life, or mortality, is unknown [8].

The study was retrospective, single-center and without external validation, so the bootstrap and cross-validation results establish internal validity only [34]. No a priori sample-size calculation was performed and the subgroup comparison was underpowered, so its null result is uninformative; with 8.25 events per variable, the individual coefficients are imprecise, and the sequential analyses used to localize the signal were exploratory and require confirmation. The univariable screen was uncorrected for multiplicity, and the discrepancy between the parametric and non-parametric comparisons of the titration event indices indicates that those group differences rest on a few extreme values. A multicenter prospective study with external validation and an adequate number of events per variable will be required.

## 5. Conclusions

The physiological response recorded during PAP titration predicts short-term adherence beyond baseline disease severity and demographic characteristics, and the signal lies principally in oxygenation: the titration lowest SpO_2_ was the only parameter to retain significance under full mutual adjustment, was independent of baseline hypoxemia, and was of almost identical magnitude, whether or not titration was technically successful. Given the limited number of outcome events and the absence of external validation, these findings are preliminary. Multicenter prospective studies combining titration physiology with patient-reported, device-related and psychosocial data are needed, to develop and validate models useful for individual risk stratification.

## Figures and Tables

**Table 1:** Comparison of demographic, clinical, polysomnographic, and PAP titration characteristics between PAP adherence and non-adherence groups (*n* = 304).

Variable	Adherence Group (*n* = 238)	Non-Adherence Group (*n* = 66)	*p*
Demographic and clinical data			
Age (years)	49.5 ± 12.2	48.7 ± 14.6	0.683
Sex, male	202 (84.9)	54 (81.8)	0.681
Body mass index (kg/m^2^)	28.8 ± 4.8	29.0 ± 5.4	0.841
Neck circumference (cm)	39.3 ± 4.3	39.1 ± 3.6	0.734
Waist-to-hip ratio	1.0 ± 0.1	1.0 ± 0.1	0.355
Epworth Sleepiness Scale	9.3 ± 5.1	8.9 ± 5.1	0.581
STOP-Bang questionnaire	4.7 ± 1.2	4.6 ± 1.2	0.278
Pittsburgh Sleep Quality Index	5.7 ± 2.4	5.5 ± 2.2	0.607
Baseline polysomnographic data			
TST (min)	339.7 ± 44.0	345.9 ± 46.7	0.334
Sleep efficiency (%)	81.2 ± 12.1	81.7 ± 12.7	0.766
AHI (events/h)	44.3 ± 25.4	39.0 ± 24.5	0.126
RDI (events/h)	49.0 ± 23.5	44.2 ± 22.6	0.130
ODI (events/h)	41.6 ± 25.8	37.9 ± 24.5	0.281
Lowest SpO_2_ (%)	77.5 ± 8.8	77.9 ± 9.0	0.720
Total arousal index	44.2 ± 21.3	39.3 ± 21.6	0.109
Stage N1 (% of TST)	30.4 ± 17.1	26.1 ± 15.3	0.051
Stage N2 (% of TST)	40.7 ± 15.5	44.9 ± 15.5	0.053
Stage N3 (% of TST)	4.3 ± 6.5	3.9 ± 5.5	0.884
Stage R (% of TST)	14.9 ± 6.5	15.8 ± 5.3	0.247
PAP titration data			
Optimal pressure (cmH_2_O)	8.7 ± 2.7	8.6 ± 2.9	0.815
Titration TST (min)	349.6 ± 48.9	346.5 ± 52.5	0.667
Titration sleep efficiency (%)	83.8 ± 12.1	81.5 ± 13.6	0.225
Titration AHI (events/h)	5.3 ± 6.0	7.8 ± 10.4	0.133
Titration RDI (events/h)	6.8 ± 5.9	9.1 ± 10.4	0.204
Titration ODI (events/h)	5.1 ± 5.5	6.8 ± 9.1	0.332
Titration lowest SpO_2_ (%)	89.4 ± 4.1	87.5 ± 5.3	0.010 *
AHI reduction rate (%)	86.8 ± 13.3	76.3 ± 29.2	0.033 *
Titration total arousal index	18.7 ± 9.7	20.9 ± 13.6	0.747
Titration respiratory arousal index	3.3 ± 4.6	5.5 ± 8.9	0.108
Titration spontaneous arousal index	12.5 ± 7.0	11.6 ± 7.9	0.156
Titration RERA index	1.5 ± 1.6	1.4 ± 1.5	0.526
Titration supine AHI (events/h)	5.5 ± 6.2	7.8 ± 10.1	0.129
Titration N1 (% of TST)	13.5 ± 7.7	15.4 ± 9.2	0.137
Titration N2 (% of TST)	51.7 ± 12.2	51.5 ± 12.8	0.874
Titration N3 (% of TST)	7.1 ± 8.2	6.1 ± 7.5	0.460
Titration REM (% of TST)	20.5 ± 7.3	18.0 ± 7.7	0.021 *

Values are presented as mean ± standard deviation or *n* (%). PAP, positive airway pressure; TST, total sleep time; AHI, apnea–hypopnea index; RDI, respiratory disturbance index; ODI, oxygen desaturation index; SpO_2_, oxygen saturation; RERA, respiratory effort-related arousal; REM, rapid eye movement. Between-group comparisons used independent-samples *t*-tests for approximately normally distributed continuous variables, the Mann–Whitney U test for the right-skewed variables (titration AHI, RDI and ODI, AHI reduction rate, the total, respiratory and spontaneous arousal indices, the RERA index, titration supine AHI, and stage N3 proportions), and χ^2^ tests for categorical variables. Welch’s correction was applied to all *t*-tests. * *p* < 0.05.

**Table 2:** Univariable logistic regression analysis of PAP titration parameters associated with PAP adherence (*n* = 304).

Variable	Unit	B ^†^	SE	OR	95% CI	AUC	*p*
Titration AHI	per 5 events/h	−0.200	0.088	0.819 *	0.690–0.972	0.560	0.022 *
Titration RDI	per 5 events/h	−0.200	0.089	0.819 *	0.687–0.975	0.551	0.025 *
Titration ODI	per 5 events/h	−0.176	0.097	0.838	0.693–1.015	0.539	0.071
Titration lowest SpO_2_	per 5%	0.425	0.147	1.530 *	1.146–2.042	0.607	0.004 *
AHI reduction rate	per 10%	0.266	0.074	1.305 *	1.128–1.509	0.586	<0.001 *
Respiratory arousal index	per 1 event/h	−0.056	0.022	0.945 *	0.905–0.987	0.565	0.011 *
Titration supine AHI	per 5 events/h	−0.187	0.087	0.829 *	0.700–0.984	0.561	0.032 *
Titration REM sleep	per 5%	0.237	0.099	1.267 *	1.044–1.538	0.596	0.017 *

Odds ratios (ORs) are presented per clinically meaningful increments as specified in the Unit column. PAP, positive airway pressure; AHI, apnea–hypopnea index; RDI, respiratory disturbance index; ODI, oxygen desaturation index; SpO_2_, oxygen saturation; REM, rapid eye movement; SE, standard error; CI, confidence interval; AUC, area under the receiver operating characteristic curve. * *p* < 0.05; ^†^ regression coefficient. Coefficients are reported on the scale of the increment given in the Unit column; the coefficient and its standard error were rescaled by the same factor, so that the Wald statistic and the *p*-value are invariant to the choice of increment.

**Table 3:** Multivariable logistic regression model for PAP adherence prediction, adjusted for baseline confounders (*n* = 304).

Variable	B ^†^	SE	OR	95% CI	*p*
Titration variables					
AHI reduction rate (per 1%)	0.012	0.013	1.012	0.987–1.038	0.361
Titration lowest SpO_2_ (per 1%)	0.111	0.043	1.117 *	1.028–1.214	0.009 *
Respiratory arousal index (per 1 event/h)	−0.004	0.040	0.996	0.922–1.076	0.919
Titration REM sleep (per 1%)	0.043	0.023	1.044	0.998–1.092	0.062
Adjustment variables					
Age (per year)	0.010	0.013	1.010	0.985–1.035	0.460
Sex, male	0.277	0.405	1.320	0.597–2.917	0.493
BMI (per 1 kg/m^2^)	−0.003	0.036	0.997	0.929–1.070	0.928
Baseline AHI (per 1 event/h)	0.013	0.009	1.013	0.995–1.031	0.164

Model apparent area under the receiver operating characteristic curve (AUC) = 0.683 (confounder-only model, 0.566; likelihood-ratio χ^2^(4) = 22.07 for the addition of the four titration variables, *p* < 0.001); optimism-corrected AUC = 0.632 (500-iteration bootstrap, 95% confidence interval [CI]: 0.563–0.700); repeated (20 × 5-fold) cross-validated AUC = 0.607 ± 0.023. Events per variable = 66/8 = 8.25. PAP, positive airway pressure; AHI, apnea–hypopnea index; SpO_2_, oxygen saturation; REM, rapid eye movement; BMI, body mass index; SE, standard error; OR, odds ratio. * *p* < 0.05; ^†^ regression coefficient.

## Data Availability

The datasets generated during and/or analyzed during the current study are available from the corresponding author on reasonable request.

## Abbreviations

The following abbreviations are used in this manuscript:

PAP	Positive airway pressure
OSA	Obstructive sleep apnea
AHI	Apnea–hypopnea index
SpO_2_	Oxygen saturation
REM	Rapid eye movement
OR	Odds ratio
AUC	Area under the curve
CPAP	Continuous positive airway pressure
BMI	Body mass index
AASM	American Academy of Sleep Medicine
RERA	Respiratory effort-related arousal
RDI	Respiratory disturbance index
ODI	Oxygen desaturation index
CI	Confidence interval
